# ERK5 Activation by Gq-Coupled Muscarinic Receptors Is Independent of Receptor Internalization and β-Arrestin Recruitment

**DOI:** 10.1371/journal.pone.0084174

**Published:** 2013-12-17

**Authors:** Guzmán Sánchez-Fernández, Sofía Cabezudo, Carlota García-Hoz, Andrew B. Tobin, Federico Mayor Jr, Catalina Ribas

**Affiliations:** 1 Departamento de Biología Molecular and Centro de Biología Molecular “Severo Ochoa”, Universidad Autónoma de Madrid, UAM-CSIC, Madrid, Spain; 2 Instituto de Investigación Sanitaria La Princesa, Madrid, Spain; 3 Medical Research Council Toxicology Unit, University of Leicester, Leicester, United Kingdom; Loyola University Chicago, Stritch School of Medicine, United States of America

## Abstract

G-protein-coupled receptors (GPCRs) are known to activate both G protein- and β-arrestin-dependent signalling cascades. The initiation of mitogen-activated protein kinase (MAPK) pathways is a key downstream event in the control of cellular functions including proliferation, differentiation, migration and apoptosis. Both G proteins and β-arrestins have been reported to mediate context-specific activation of ERK1/2, p38 and JNK MAPKs. Recently, the activation of ERK5 MAPK by Gq-coupled receptors has been described to involve a direct interaction between Gαq and two novel effectors, PKCζ and MEK5. However, the possible contribution of β-arrestin towards this pathway has not yet been addressed. In the present work we sought to investigate the role of receptor internalization processes and β-arrestin recruitment in the activation of ERK5 by Gq-coupled GPCRs. Our results show that ERK5 activation is independent of M1 or M3 muscarinic receptor internalization. Furthermore, we demonstrate that phosphorylation-deficient muscarinic M1 and M3 receptors are still able to fully activate the ERK5 pathway, despite their reported inability to recruit β-arrestins. Indeed, the overexpression of Gαq, but not that of β-arrestin1 or β-arrestin2, was found to potently enhance ERK5 activation by GPCRs, whereas silencing of β-arrestin2 expression did not affect the activation of this pathway. Finally, we show that a β-arrestin-biased mutant form of angiotensin II (SII; Sar1-Ile4-Ile8 AngII) failed to promote ERK5 phosphorylation in primary cardiac fibroblasts, as compared to the natural ligand. Overall, this study shows that the activation of ERK5 MAPK by model Gq-coupled GPCRs does not depend on receptor internalization, β-arrestin recruitment or receptor phosphorylation but rather is dependent on Gαq-signalling.

## Introduction

G-protein-coupled receptors (GPCRs) are the largest and most versatile family of transmembrane receptor in mammalian cells [1]. The classical model for GPCR action involves G protein-mediated signal transduction and progressive desensitisation mediated by GPCR kinases (GRKs) and β-arrestins. GRKs are known to phosphorylate the internal loops of activated receptors thus creating recognition sites for high-affinity binding of β-arrestins. These proteins uncouple receptors from heterotrimeric G proteins and promote receptor internalization [2]. However, over the last few years there has been a new appreciation of the capacity of β-arrestins to act as multifunctional adaptor proteins that have the ability to couple GPCRs to numerous signalling components such as mitogen-activated protein kinases (MAPKs), Src, nuclear factor-κB (NF-κB) and phosphoinositide 3-kinase (PI3K) [3]. The actions of G proteins and β-arrestins are diverse and are often spatially and temporarily segregated. For instance, the contribution of G protein/β-arrestin towards the activation of ERK1/2 by angiotensin II is additive but differs in kinetics, timeframe and localisation [4]. Upon stimulation of the AT_1A_ receptor, ERK1/2 is immediately, but transiently, activated via the G protein-dependent pathway, whereas β-arrestin2-mediated ERK1/2 activation is relatively slow but persistent. Interestingly, G protein-activated ERK1/2 is found in the nucleus whereas it localises in the cytoplasm upon β-arrestin activation. Additionally, mutational studies have determined the independence of both pathways with mutant versions of the angiotensin AT_1A_ receptor that are unable to couple to G proteins while still able to recruit β-arrestins [5,6].

Activated Gq/11-coupled GPCRs trigger different signals that have been implicated in the control of MAPK pathways, including ERK1/2, p38, JNK and ERK5 cascades. Both Gq-dependent and β-arrestin-dependent mechanisms have been described for the activation of MAPKs, except from the most recently described ERK5 [3]. The GPCR-initiated pathway leading to ERK5 does not involve the classical routes (Ras, Rho, Rac, and/or Cdc42) for the activation of MAPK by GPCR [7]. Recently, our group described the molecular mechanism for the activation of ERK5 by the Gq-coupled M1 muscarinic receptor in epithelial cells [8]. This pathway did not require PLCβ activity and involved the atypical protein kinase C zeta (PKCζ) and the MAPKK MEK5 as two novel effectors of Gαq. Such process was found to be conserved in the heart and to be important in the development of angiotensin II-induced hypertrophic programmes *in vivo* [9]. Thus, it was shown that Gq-coupled muscarinic and angiotensin II receptors activate ERK5 through a similar mechanism but the contribution of β-arrestin towards the novel pathway was not addressed. Therefore, in the present study we have aimed to establish relative contributions of Gαq- versus β-arrestin-dependent signalling in the activation of ERK5.

## Materials and Methods

### Ethics statement

Mice for the isolation of primary cultures were maintained under pathogen-free conditions, and all of the experiments were performed in accordance with the guidelines of the European Convention for the Protection of Vertebrate Animals used for Experimental and Other Scientific Purposes (Directive 86/609/ EEC) and with the authorization of the Bioethical Committee of the Universidad Autónoma de Madrid (CEI-21-440).

### Materials

The cDNA of Gαq was kindly provided by Dr. Anna Aragay (CSIC Barcelona, Spain). β-arrestin1-FLAG was provided by V. Gurevich (Vanderbilt University, Nashville, USA) and β-arrestin2-GFP was a gift from Dr. J.L. Benovic (Kimmel Cancer Center, Philadelphia, USA). The cDNA encoding HA-ERK5 has been previously described [8]. NIH-3T3 fibroblasts expressing approximately 20,000 human M1 muscarinic receptors per cell, designated NIH-3T3-M1 cells, were kindly provided by Dr. J.S. Gutkind (NIH, Bethesda, MD, USA). CHO-M3 internalization-deficient cells expressing a mutant version of the muscarinic M3 receptor that cannot internalize (motif SASS to AAAA) were previously characterised [10]. CHO-M3 *wild-type*, CHO-M3 phospho-deficient, CHO-M1 *wild-type* and CHO-M1 phospho-deficient cells were previously generated [11] in the Flp-In genetic background to guarantee equal expression of receptors amongst the different cell lines [12]. Culture media and Lipofectamine were from Life Technologies Inc. (Gaithersburg, MD, USA). The monoclonal antibody against Gαq was from Abnova. Rabbit anti-phospho-ERK5 antibody (p-Thr218/p-Tyr220) was purchased from Invitrogen. Anti-ERK5 and anti phospho-ERK1/2 were from Cell Signalling. Anti-ERK1 and anti-ERK2 antibodies were from Santa Cruz. Anti-alpha tubulin and anti-FLAG were from Sigma. Anti-GFP was from Roche. Anti-clathrin heavy chain was from BD Transduction. Carbachol, acetylcholine and angiotensin II were from Sigma, and SII (Sar1-Ile4-Ile8 Ang II) was from Bachem. All other reagents were of the highest commercially available grades.

### Cell line culture and treatment

NIH-3T3-Ml and CHO cells were maintained in Dulbecco’s modified Eagle’s medium (DMEM) supplemented with 10% (v/v) Newborn serum (Gibco) or fetal bovine serum (Sigm-Aldrich, St. Louis, MO, USA), respectively, at 37°C in a humidified 5% CO_2_ atmosphere. The desired cell type was stimulated with different muscarinic receptor ligands (carbachol or acetylcholine) at 37°C in serum-free DMEM media, at the specified doses and during the indicated time periods. It is noteworthy that acetylcholine and carbachol are chemically equivalent species and, in our hands, have been proven interchangeable in their ability to promote ERK5 activation. The cells were serum-starved for 5-6 h before ligand addition to minimise basal kinase activity. CHO cells (70-80% confluent monolayers in 60 mm dishes) were transiently transfected with the desired combinations of cDNA constructs using the Lipofectamine/Plus method, following manufacturer’s instructions. Empty vector was added to keep the total amount of DNA per dish constant. Assays were performed 24h after transfection. Transient expression of the desired proteins was confirmed by immunoblot analysis of whole-cell lysates using specific antisera, as described below.

Primary cultures of neonatal fibroblasts were prepared from C57BL/6J mice by dissociation of neonatal (1-2 days) hearts. After excised, hearts were kept in Ca^++^-free medium at 4°C and were mechanically disaggregated using a sterile scalpel blade. For the chemical disaggregation the tissue was transferred to a culture flask (T25) and kept in trypsin (0.05%) and type II collagenase (Worthington) for 15min at 37°C after which the supernatant was collected and mixed with medium with calcium to stop the reaction. The macroscopical tissue fragments that remained in the flask were further disaggregation in up to 5 trypsinization steps. Each medium collected was centrifuged (5min, 1500 r.p.m.), resuspended in medium supplemented with 10% FBS and kept at 37°C. Cells were plated and incubated for 2h to allow for non-myocytes to attach, after which time the medium containing cardiac myocytes was discarded. Cardiac fibroblasts were expanded in DMEM supplemented with 10% FBS and passed twice before the assay to remove contamination by endothelial cells. Cells were stimulated with angiotensin II (100 nM) or SII (Sar1-Ile4-Ile8 (SII) Ang II, 10µM) as previously described [13] in serum-free media, during the indicated time periods. In order to minimise ERK5 basal kinase activity before ligand addition, cells were serum-starved for 5-6 h. Alternatively, to assess ERK1/2 activation cells were starved for 16h, treated with specific MEK1/2 inhibitor (PD98059, 50µM, Cell signalling) for 30 minutes, washed in PBS and let stand in serum-free medium for another 30 minutes before ligand stimulation.

### Radioligand binding assays

Expression of cell surface muscarinic receptors after exposure to carbachol for different times was determined as described previously [14] except that saturating concentrations (0.5 nM) of the muscarinic antagonist [^3^H]-NMS were used in incubations with whole cells for 120 min at 4°C.

### siRNA-mediated protein silencing

NIH-3T3-M1 cells were reverse-transfected using siRNA specific oligos for clathrin (mouse CLTC, heavy chain, on-target plus SMARTpool), or β-arrestin2 (mouse Arrb2, on-target plus SMARTpool) purchased from Dharmacon (Roche, Palo Alto, CA). Scrambled oligos were purchased from Ambion to serve as negative control. siRNAs were transfected using DharmaFECT 1 reagent (Dharmacon) to reach a final concentrations of 100/250nM. Specific antibodies were utilised to assess effective knockdown by Western blot.

### Determination of MAPK stimulation

Activation of endogenous ERK5 was detected in NIH-3T3-M1 cells and in primary cardiac fibroblasts by Western blot analysis of cell lysates with a phosphospecific anti-ERK5 antibody. In CHO cell lines, due to the low ERK5 endogenous levels, HA-tagged ERK5 was transfected and immunoprecipitated with anti-HA agarose beads (Santa Cruz). Immunoprecipitates were washed 6 times in lysis buffer (50 mM Tris-HCl, 150 mM NaCl, 1% (w/v) Nonidet P-40, 0.25% (w/v) sodium deoxycholate, 1 mM EGTA, 1 mM NaF, supplemented with 1 mM sodium orthovanadate plus a mixture of protease inhibitors) at 4°C. Lysates were resolved by 8% SDS-PAGE and subjected to immunoblot analysis as previously described [8] and the activation state of ERK5 was measured. To obtain cell lysates, cells were washed with ice-cold PBS-buffer plus 1mM sodium orthovanadate and subsequently solubilised in lysis buffer (50mM Tris-HCl, 150mM NaCl, 1% (w/v) Nonidet P-40, 0.25% (w/v) sodium deoxycholate, 1mM EGTA, 1mM NaF, supplemented with 1 mM sodium orthovanadate plus a mixture of protease inhibitors). Lysates were resolved by 6-10% SDS-PAGE and subjected to immunoblot analysis. All blots were developed using the chemiluminescence method (ECL, Amersham Pharmacia Biotech, UK). Bands were quantified by laser-scanner densitometry and the amount of phospho-ERK5 protein normalised to the amount of the total ERK5 protein, as assessed by the specific antibodies. The activation of the ERK1/2 pathway was determined as previously reported [8]. Statistical analysis was performed using the two-tailed Student’s T-test, as indicated.

## Results

Since β-arrestin has been recently proposed as an alternative signalling node to heterotrimeric G proteins, it is essential to ascertain whether a known GPCR-initiated pathway like ERK5 is equally or differentially activated by these two signalling players.

### ERK5 activation by Gq-coupled GPCR does not require receptor internalization

In order to facilitate the study of the mechanistic basis of GPCR-ERK5 activation we initially utilised different cell lines stably expressing Gq-coupled (muscarinic) receptors. This has been widely proven to be the most successful experimental approach to study ERK5 phosphorylation using the currently available detection methods. In particular, the NIH-3T3 cell line that stably overexpresses the human M1 muscarinic receptor has been extensively used to detect endogenous ERK5 activation by us [8,9] and others [7].

**Figure 1:**
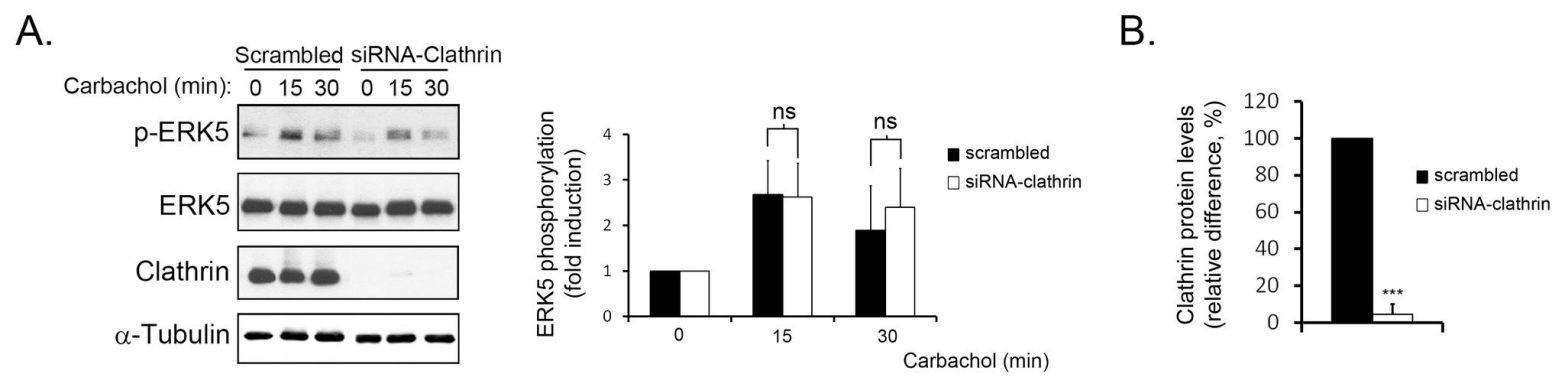
Gq-coupled muscarinic receptor-induced activation of the ERK5 pathway is clathrin-independent. NIH-3T3 cells stably overexpressing the muscarinic M1 receptor (NIH-3T3-M1 cells) were transfected with siRNA oligonucleotides targeting clathrin heavy chain or non-targeting scrambled oligonucleotides (250nM) as detailed in the Materials and Methods section. After 72h of incubation, cells were serum-starved for 5h and challenged with carbachol (10µM) for the indicated times. (A) Endogenous ERK5 phosphorylation was assessed in cell lysates with a phospho-ERK5 specific antibody. Data (mean +/- SEM of 3 independent experiments) were normalised using ERK5 as loading control and expressed as fold-induction over basal conditions. (B) Clathrin expression levels were also determined and quantified to estimate the overall efficiency of protein silencing. Data (mean +/- SEM of 3 independent experiments) were normalised using α-tubulin as loading control and expressed as the relative difference (%) to control.

**Figure 2:**
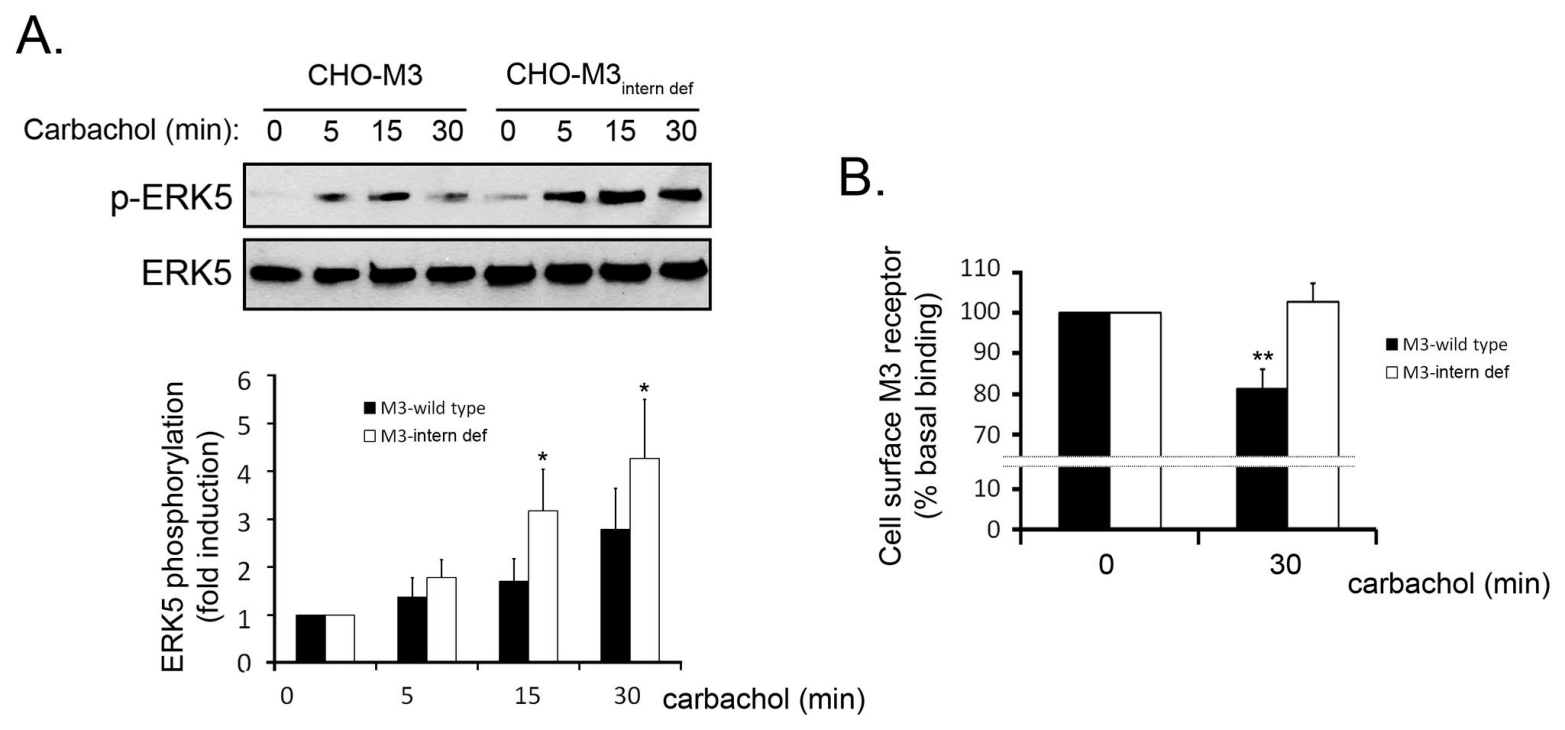
Gq-coupled muscarinic receptor-induced activation of the ERK5 pathway does not require receptor internalization. (A) CHO cells stably overexpressing *wild-type* muscarinic M3 receptor or internalization-deficient M3 receptor (SASS motif mutant, characterised in [10]) were transfected with HA-ERK5. Twenty-four hours after transfection, cells were serum-starved for 2h and stimulated with carbachol (10µM). HA-ERK5 was immunoprecipitated with an anti-HA agarose-conjugated antibody as detailed in the Materials and Methods section. ERK5 phosphorylation was assessed in the immunoprecipitate using a phosphospecific antibody. Data (mean +/- SEM of 3 independent experiments) were normalised using HA-ERK5 as loading control and expressed as fold-induction over basal conditions (*p<0.05, two-tailed T-test). (B) Quantification of cell surface receptor density was performed through [^3^H]-NMS binding at 4°C. The two cell types utilised in (A) were serum-starved for 2h and stimulated with carbachol (100µM) for 30 minutes. Data were normalised to unspecific binding (atropine treatment) and binding percentage was expressed as the mean +/- SEM of 3 independent experiments.

Upon activation, GPCR internalize in a process that will lead to recycling or degradation of the receptor. These receptor trafficking processes have been reported to also provide alternative ways for signalling propagation via β-arrestin [3]. Since β-arrestin recruitment leads to GPCR internalization in a process where clathrin plays a key role [2,15], we determined the activation of the ERK5 pathway in a clathrin knock-down scenario. Thus, NIH-3T3-M1 cells were pre-treated with scrambled siRNA or clathrin-targeting oligonucleotides and stimulated with the muscarinic agonist carbachol for 30 minutes (Figure 1A). Results indicated that ERK5 phosphorylation was unaffected in the absence of clathrin (95% knock-down efficiency, as quantified in Figure 1B) thus suggesting that clathrin-mediated M1 receptor internalization is likely not required for this process. Although the primary internalization mechanisms for muscarinic Gq-coupled receptors are clathrin-dependent [16,17], alternative processes have also been reported [18]. To assess the contribution of clathrin-independent mechanisms towards the ERK5 pathway, we utilised a CHO cell line that overexpresses an internalization-deficient mutant M3 muscarinic receptor. This receptor harbours several mutations on the third internal loop (motif SASS to AAAA) that prevent trafficking into endosomes [10]. We transfected HA-tagged ERK5 into CHO-M3 and CHO-M3-internalization-deficient cells and stimulated with carbachol at different times. Interestingly, we detected an increased and sustained activation of ERK5 by the internalization-deficient mutant compared to control (Figure 2A). The inability of the activated mutant receptor to internalize was also confirmed (Figure 2B). Collectively, these findings suggest that ERK5 activation by Gq-coupled muscarinic receptors occurs independently of receptor internalization. 

### ERK5 activation by Gq-coupled GPCR does not involve receptor phosphorylation or β-arrestin recruitment

**Figure 3:**
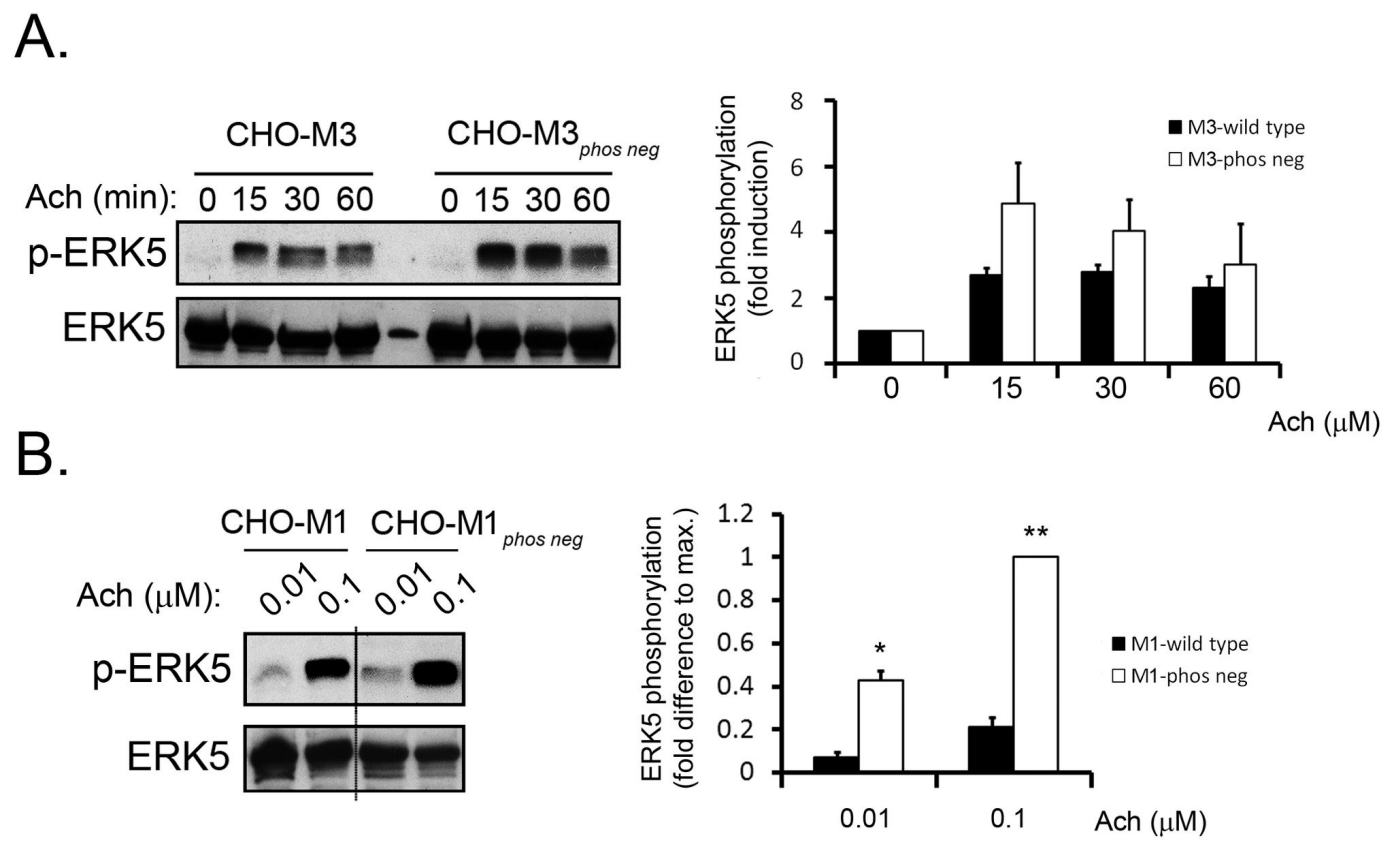
Gq-coupled muscarinic receptor-induced activation of the ERK5 pathway does not require receptor phosphorylation. Different stable lines of CHO cells (previously characterised in [11]) that stably overexpress *wild-type* or phosphorylation-deficient muscarinic M3 receptor (A) and *wild-type* or phosphorylation-deficient muscarinic M1 receptor (B) were utilised. All cell lines were transfected with HA-ERK5. Twenty-four hours after transfection, cells were serum-starved for 2h and stimulated with acetylcholine (100µM) for the indicated times (A) or incubated for 15min with acetylcholine at various concentrations (B). ERK5 phosphorylation was assessed as in Figure 2. Data (mean +/- SD of 2-3 independent experiments) were normalised using HA-ERK5 as loading control and expressed as fold-induction over basal conditions or over maximum activation (*p<0.05, **p<0.005; two-tailed T-test).

β-arrestin is generally known to be recruited to GPCRs through a mechanism dependent the phosphorylation of the receptor. Thus, the translocation of arrestins to the plasma membrane occurs through the recognition of phosphorylation sites on the internal loops and C-terminal tails of receptors. Since ERK5 activation does not seem to depend on receptor internalization, if β-arrestin participates in this pathway, this would not occur in the endosomes. However, it remained the possibility that β-arrestin could initiate pathways leading to ERK5 activation from the plasma membrane. In order to ascertain this, we utilised a CHO cell line overexpressing a phospho-deficient muscarinic M3 mutant receptor. This receptor, in which all the phosphorylatable serines within the internal loops are mutated to alanine, has been described to have a significantly reduced ability to recruit β-arrestins in the same cell line used in our study but to be otherwise functional [11]. Notably, the amplitude of time-dependent ERK5 phosphorylation upon acetylcholine treatment was augmented by the phosphodeficient M3 receptor compared to the wild-type form of the receptor (Figure 3A). To further confirm this, we used a similar phosphodeficient version of the M1 receptor and stimulated for 15 minutes with sub-maximal doses of acetylcholine in order to highlight the differences in ERK5 activation by *wild-type* or mutant receptor. Indeed, we observed a clear enhancement of ERK5 activation by the phosphodeficient mutant M1 receptor (Figure 3B). Taken together, these data suggest that receptor phosphorylation and the subsequent recruitment of β-arrestins are not involved in ERK5 activation by Gq-coupled muscarinic GPCRs. 

### ERK5 activation by GPCR is Gαq-biased

**Figure 4:**
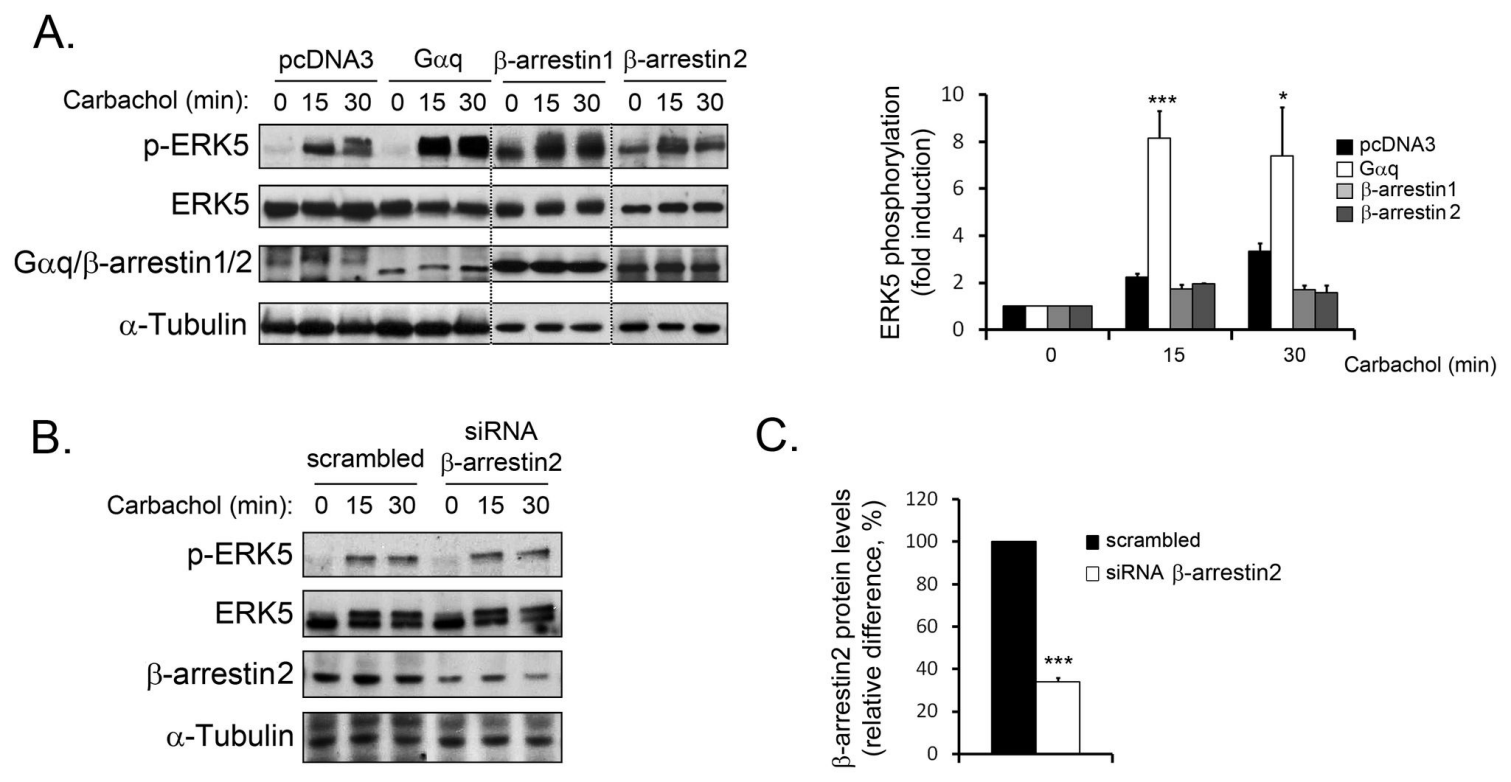
β-arrestins are not involved in ERK5 activation by Gq-coupled muscarinic receptors. (A) CHO cells stably expressing *wild-type* muscarinic M3 receptor (CHO-M3 cells) were transfected with cDNAs encoding for HA-ERK5 and either pcDNA3, Gαq, β-arrestin1-Flag or β-arrestin2-GFP. Twenty-four hours after transfection, cells were serum-starved for 2h and stimulated with carbachol (10µM) for the indicated times. ERK5 phosphorylation was assessed as in Figure 2. Data (mean +/- SEM of 3 independent experiments) were normalised using HA-ERK5 as loading control and expressed as fold-induction over basal conditions (*p<0.05, **p<0.005, two-tailed T-test). Gαq, β-arrestin1-Flag and β-arrestin2-GFP, and α-tubulin expression levels were assessed with specific antibodies. (B) NIH-3T3-M1 cells were transfected with siRNA oligonucleotides targeting β-arrestin2 or non-targeting scrambled oligonucleotides (100nM) as detailed in the Materials and Methods section. After 72h of incubation, cells were serum-starved for 5h and challenged with carbachol (10µM) for the indicated times. Endogenous ERK5 phosphorylation was assessed in cell lysates with a phospho-ERK5 specific antibody. Total ERK5 appears as a double band corresponding to basal (lower) and hyper-phosphorylated (upper) kinase. A representative blot for 3 independent experiments with similar results is shown. (C) β-arrestin2 expression levels were also determined and quantified to estimate the overall efficiency of protein silencing. Data (mean +/- SEM of 3 independent experiments) were normalised using α-tubulin as loading control and expressed as the relative difference (%) to β-arrestin2 protein levels in scrambled siRNA-treated cells.

**Figure 5:**
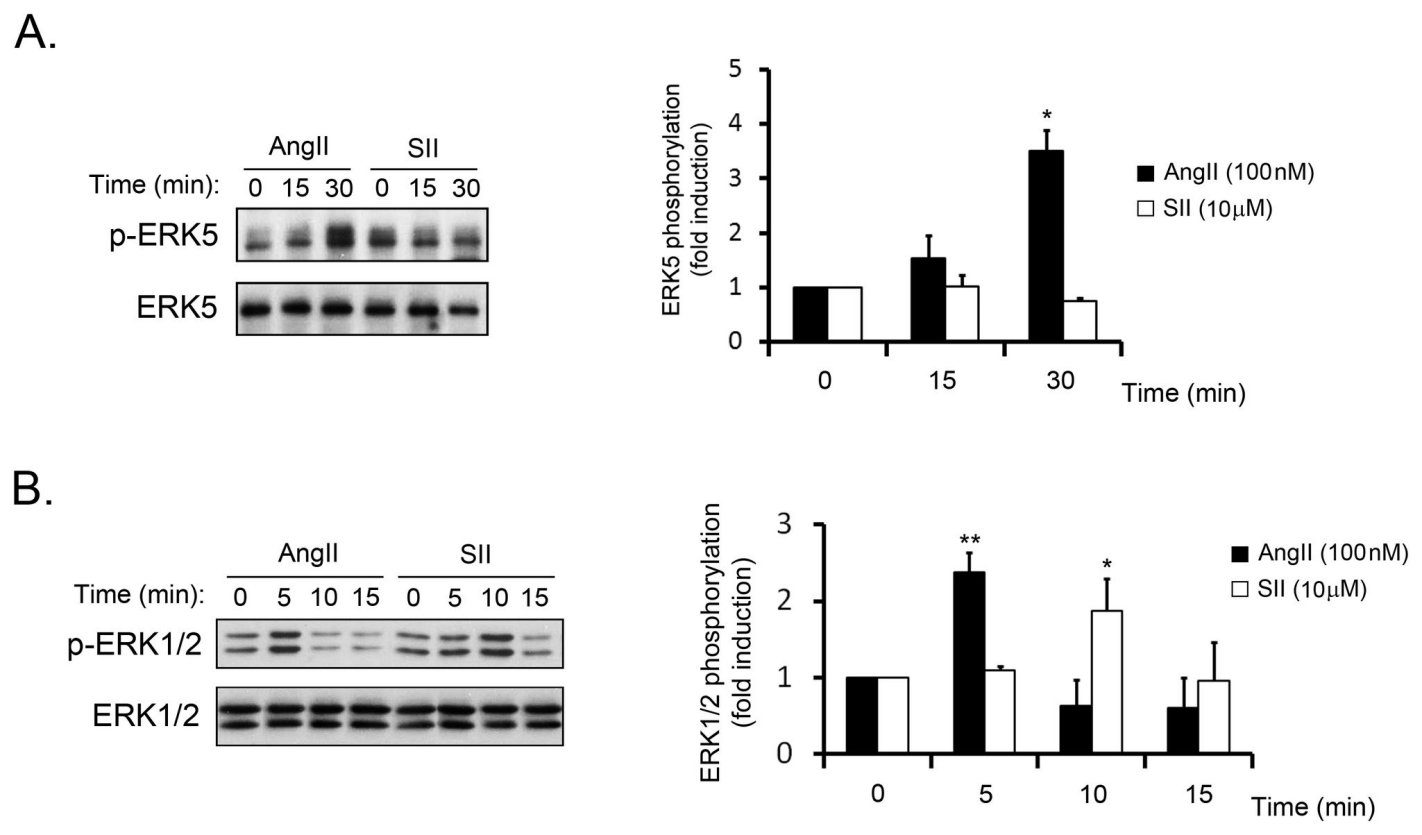
ERK5 is not activated by a β-arrestin-biased agonist in cardiac fibroblasts. Neonatal mouse cardiac fibroblasts were challenged with angiotensin (AngII, 100nM) or Sar1-Ile4-Ile8-AngII (SII, 10µM), for the indicated times. Endogenous ERK5 (A) or ERK1/2 (B) activation was determined in cell lysates with phospho-specific antibodies. Data (mean +/- SEM of 3 independent experiments) were normalised using total ERK5 or ERK1/2 as loading controls and expressed as fold induction compared with the absence of agonist (*p<0.05, **p<0.005, two tailed T-test).

The data above indicated that Gq-initiated signalling is the key participant in ERK5 activation by muscarinic Gq-coupled GPCR. We sought to confirm this notion by studying the activation of ERK5 in a Gαq-enriched cell population. In these circumstances, Gq-dependent pathways would be potentiated whereas β-arrestin-dependent pathways would either remain unaltered or be downregulated as a result of a functional competition. Interestingly, the activation of ERK5 in CHO-M3 cells overexpressing Gαq was greatly enhanced compared to control (Figure 4A). On the contrary, the overexpression of either non-visual β-arrestin, β-arrestin1&2) did not favour ERK5 activation. Coincidently, we demonstrate that silencing of β-arrestin2 protein expression in NIH-3T3-M1 cells did not affect carbachol-induced ERK5 activation (Figure 4B-C). These results confirm that Gαq activation is the predominant event in the ERK5 pathway whereas β-arrestins are not involved. In order to further establish the Gαq-biased nature of this pathway we utilised the angiotensin receptor system, which has been reported to activate ERK5 through a similar mechanism to that described for Gq-coupled muscarinic receptors [9]. To this purpose, we utilised a synthetic angiotensin II ligand (Sar1-Ile4-Ile8 (SII) AngII) that displays biased properties towards β-arrestin pathways and has been previously reported to activate MAPK pathways in certain contexts [13]. As shown in Figure 5A, the ERK5 pathway is not activated by SII as compared to the natural ligand angiotensin II in primary cardiac fibroblasts. Interestingly, the β-arrestin-biased ligand was able to promote the activation of ERK1/2, which indicates the occurrence of alternative mechanisms for the activation of different MAPK pathways (Figure 5B). Collectively, these results suggest that the mechanism for ERK5 activation by model Gq-coupled receptors is Gαq-biased.

## Discussion

The biological functions of GPCR are being currently revised in the light of novel findings on the ability of β-arrestins to promote signalling independently of G proteins. For instance, the activation of ERK1/2 by angiotensin II has been reported as dual; one initial and rapid stage (after 2 minutes) driven by Gαq and a later and more sustained activation (after 10 minutes) driven by arrestins after receptor internalization [4]. Since ERK5 phosphorylation by Gq-coupled GPCR occurs at a relatively late stage after receptor activation (usually peaking around 15/30 minutes [8]) it was tempting to suggest an involvement of β-arrestin through mechanisms dependent on receptor phosphorylation and internalization. In the same lines, neutrophin-induced ERK5 activation in neurons has been shown to require the internalization of the p62/PKCζ complex [19]. However, our results clearly show that activation of ERK5 by muscarinic M1/M3 receptors does not seem to involve receptor internalization, which is one of the mechanisms for β-arrestin to initiate signalling. The possibility remained that a β-arrestin-mediated event occurred at the plasma membrane. However, we found that phosphorylation-deficient muscarinic receptors that cannot recruit β-arrestin or internalize [11] managed to enhance ERK5 activation in comparison to wild-type receptors. Additionally, a β-arrestin-biased angiotensin receptor ligand failed to activate ERK5 in cardiac fibroblasts. On the contrary, we demonstrate that the overexpression of Gαq, but not that of β-arrestin1 or 2, greatly potentiates ERK5 activation by Gq-coupled GPCR whereas this cascade is not affected by β-arrestin2 knockdown, thus suggesting that the activation of the ERK5 pathway is independent of β-arrestin and displays Gαq-biased properties. Thus, we propose that the unique properties of the Gαq/ERK5 pathway, where Gαq acts as a scaffold [8] (a role usually assigned to β-arrestin), could represent a possible common mechanism for Gαq-bias. The activation of several families of MAPK by GPCRs have been shown to require β-arrestin-signalling to some degree and in certain contexts, either through direct interaction between MAPK cascade components and β-arrestin [20–22], or being activated downstream β-arrestin-initiated pathways [3]. Alternatively, our results on the Gαq-biased nature of the ERK5 pathway are consistent with reports for Gq-coupled muscarinic receptors [14], showing that the activation of the ERK1/2 family is primarily Gαq- and not β-arrestin-dependent. 

The determination of the biased properties of some agonist/receptor systems is crucial to understand the functional diversity of GPCRs involved in pathological contexts. A specific new class of biased drugs against the AT1 type II receptor has been developed with promising results in a cardiovascular setting [23]. In these lines, it is tempting to suggest that the Gαq-biased properties of the ERK5 pathway could further our understanding of angiotensin II-promoted cardiac hypertrophy, where this pathway was recently shown to be relevant [9], and potentially contribute to the fine tuning of therapeutic interventions. 
